# Genome-scale metabolic model of the rat liver predicts effects of diet restriction

**DOI:** 10.1038/s41598-019-46245-1

**Published:** 2019-07-08

**Authors:** Priyanka Baloni, Vineet Sangar, James T. Yurkovich, Max Robinson, Scott Taylor, Christine M. Karbowski, Hisham K. Hamadeh, Yudong D. He, Nathan D. Price

**Affiliations:** 1 Institute for Systems Biology, Seattle, WA United States of America; 2 Department of Comparative Biology and Safety Sciences, Amgen Inc., Thousand Oaks, CA United States of America; 3 Present Address: Genmab, Princeton, NJ United States of America

**Keywords:** Biochemical reaction networks, Computational models, Data integration, Biochemical networks, Systems analysis

## Abstract

Mapping network analysis in cells and tissues can provide insights into metabolic adaptations to changes in external environment, pathological conditions, and nutrient deprivation. Here, we reconstructed a genome-scale metabolic network of the rat liver that will allow for exploration of systems-level physiology. The resulting *in silico* model (iRatLiver) contains 1,882 reactions, 1,448 metabolites, and 994 metabolic genes. We then used this model to characterize the response of the liver’s energy metabolism to a controlled perturbation in diet. Transcriptomics data were collected from the livers of Sprague Dawley rats at 4 or 14 days of being subjected to 15%, 30%, or 60% diet restriction. These data were integrated with the iRatLiver model to generate condition-specific metabolic models, allowing us to explore network differences under each condition. We observed different pathway usage between early and late time points. Network analysis identified several highly connected “hub” genes (*Pklr*, *Hadha*, *Tkt*, *Pgm1*, *Tpi1*, and *Eno3*) that showed differing trends between early and late time points. Taken together, our results suggest that the liver’s response varied with short- and long-term diet restriction. More broadly, we anticipate that the iRatLiver model can be exploited further to study metabolic changes in the liver under other conditions such as drug treatment, infection, and disease.

## Introduction

Metabolic adaptation is critical for the ability of cells to maintain homeostasis following a physiological change. One of the more important organs for regulating homeostasis is the liver, which plays a primary role in detoxification, protein synthesis, and nutrient regulation^1,2^. Homeostatic regulation in hepatocytes involves many metabolic processes spanning interconnected pathways, requiring a systems approach to provide mechanistic insight. GEnome-scale metabolic Models (GEMs) provide one such systems biology framework for the quantitative interrogation of metabolic capabilities across diverse conditions^3^. GEMs detail the connectivity of the metabolic network through reaction stoichiometries, allowing for systems-level computation of reaction fluxes in response to genetic or environmental perturbations^4,5^.

The scope of GEMs has been iteratively expanded to include additional pathways and physiological information, including various -omics data^6–8^. Notably, the integration of transcriptomics data has allowed for the construction of condition-specific models^9–12^. The global human metabolic network reconstruction^13–15^ paved the way for the use of GEMs to explore clinical applications and resulted in many cell- and tissue-specific GEMs^16–18^. Several of these tissue-specific models have been used to study human physiology^19–22^ and pharmacological targets^23–25^. The hepatocyte has been the focus of several cell-specific modeling efforts. In 2010, two GEMs of the human hepatocyte were published simultaneously^26,27^ to understand diverse physiological liver functions; one of these models^26^ was later used to simulate metabolic phenotypes resulting from inborn errors of metabolism^28^. A later GEM, iHepatocytes^22,23^, was used to predict serine deficiency in patients with non-alcoholic fatty liver disease and to identify genes that are potential therapeutic targets for treatment of non-alcoholic steatohepatitis^29^.

While these cell-specific models have been used to understand pathophysiology in humans, the limitations associated with perturbation experiments in humans have limited their utility in translation research. Thus, organisms like Sprague Dawley rats (Rattus norvegicus) – small in size, easy to handle, high rate of reproduction, and similar physiology to humans – have been used as a primary model organism to study toxicology^30^ and to model aspects of human physiology^30^. Studying these diverse metabolic processes of the liver provides important insights into the physiological response to pharmacological interventions^31^. One of the well-studied physiological responses in the liver is due to a change in nutritional status, such as over-fed or starvation conditions^32–34^. During a well-fed state, the liver stores excess glucose as glycogen, which is then converted back to glucose during glucose deprivation. Understanding the metabolic regulatory responses to such perturbations in various physiological models represents an important open area of research.

Here, we present the *in silico* investigation of the metabolic effects of different diet-restriction patterns as interpreted through our reconstruction of a liver-specific GEM of the rat (iRatLiver). We generated genome-wide transcriptomics data of the rat liver to assess the response to different feeding patterns. These data were then integrated with iRatLiver to generate condition-specific models that were used to simulate changes in metabolic pathway usage as a result of the dietary changes. More broadly, we anticipate that this model can be a useful resource for toxicological and biomedical research, with its comparability back to the human reconstructions.

## Results

### Constructing the iRatLiver GEM

The first major step of this project was to reconstruct the metabolic network of the rat liver^35^. Because we are ultimately interested in studying human physiology, we used an existing GEM of the human liver^16^ as a starting point for the homology-based reconstruction of rat liver metabolism; see Methods for details regarding the reconstruction process. The resulting model, iRatLiver, comprises 1882 reactions, 1448 metabolites, 994 genes, 7 compartments (cytoplasm, lysosome, mitochondria, nucleus, endoplasmic reticulum, peroxisome, and extracellular space), and 82 metabolic subsystems (Supplementary Fig. 1); these subsystems were assigned according to the BiGG Models database^36^. We validated the iRatLiver model by comparing the predicted doubling time with literature values; the predicted doubling time of 16.3 hours was consistent with the reported doubling time of 16.9 hours of rat hepatocytes in cell culture^37^. Further, we tested the model’s ability to perform liver-specific functions (gluconeogenesis, triglyceride synthesis, amino acid degradation, and ammonia and ethanol detoxification) as previously reported^16^ (see Data S3 (Table S4) for simulation results). The iRatLiver model is provided in Data S1; an SBML version of the model is provided in Data S2.

There are several differences between human and rat metabolism^38–40^, most notably the existence of several enzymes that are functional in rats but are present only as pseudogenes in humans (Table 1). We compared the enzymes in rat and human and identified these unique enzymes that are functional in rats (see Methods section). The human L-threonine 3-dehydrogenase gene is an expressed pseudogene^41–44^; whereas it is functional in rats, suggesting that these differences should be taken into consideration for pharmacokinetic studies. Humans and rat also differ in their ability to metabolize uric acid; rats have a functional uric acid oxidase, whereas humans have a loss of uricase activity^45^.

**Table 1:** Enzymes present in the rat liver but not in the human liver.

Gene symbol	Enzyme	Enzyme name	Reaction formula	Pathway	Ref.
Tdh	1.1.1.103	L-threonine 3-dehydrogenase	L-threonine + NAD+ = L-2-amino-3-oxobutanoate + NADH + H+	Glycine, serine and threonine metabolism	^41^
Gulo	1.1.3.8	L-gulonolactone oxidase	L-gulono-1,4-lactone + O_2_ = L-ascorbate + H_2_O_2_	Ascorbate and aldarate metabolism	^43^
Cmah	1.14.18.2	CMP-N-acetylneuraminate monooxygenase	CMP-N-acetylneuraminate + 2 ferrocytochrome b5 + O_2_ + 2 H+ = CMP-N-glycoloylneuraminate + 2 ferricytochrome b5 + H_2_O	Amino sugar and nucleotide sugar metabolism	^79^
Uox	1.7.3.3	uric acid oxidase	urate + O_2_ + H_2_O = 5-hydroxyisourate + H_2_O_2_	Purine metabolism	^45^
Ggta1	2.4.1.87	N-acetyllactosaminide 3-alpha-galactosyltransferase	UDP-alpha-D-galactose + beta-D-galactosyl-(1->4)-beta-N-acetyl-D-glucosaminyl-R = UDP + alpha-D-galactosyl-(1->3)-beta-D-galactosyl-(1->4)-beta-N-acetylglucosaminyl-R (where R can be OH, an oligosaccharide or a glycoconjugate)	Glycosphingolipid biosynthesis - lacto and neolacto series	^80^
Art2b	3.2.2.5	NAD glycohydrolase	NAD+ + H_2_O = ADP-D-ribose + nicotinamide	Nicotinate and nicotinamide metabolism	^81, 82^
RGD1309350	3.5.2.17	hydroxyisourate hydrolase	5-hydroxyisourate + H_2_O = 5-hydroxy-2-oxo-4-ureido-2,5-dihydro-1H-imidazole-5-carboxylate	Purine metabolism	^42^
LOC688286	4.1.2.48	low-specificity L-threonine aldolase	L-threonine = glycine + acetaldehyde	Glycine, serine and threonine metabolism	^83^

We compared the iRatLiver model to an existing tissue-specific model of the human liver, liverCADRE^16^, observing differences in several subsystems: vitamin C metabolism, vitamin B2 metabolism, fatty acid oxidation, tryptophan metabolism, and the pentose phosphate pathway (Supplementary Fig. 1A). Recently, Papin and colleagues reported iRno, a GEM of rat^30^ that was used for biomarker prediction. A model of global rat metabolism, iRno encompasses all reactions in the organism rather than tissue-specific content and thus contains more reactions and metabolites than does iRatLiver. We compared our liver-specific model to the global iRno model (Supplementary Fig. 1B), finding fewer dead-end metabolites (five and 679, respectively), a result not unexpected due to the differing scope between the models (Supplementary Fig. 1), i.e. a global reconstruction vs. a reconstruction more tailored to a particular organ. There have been several subsequent iterations of iRno focusing on various aspects of rat physiology^46–48^.

### Validating iRatLiver predictions in diet-restriction conditions

**Figure 1:**
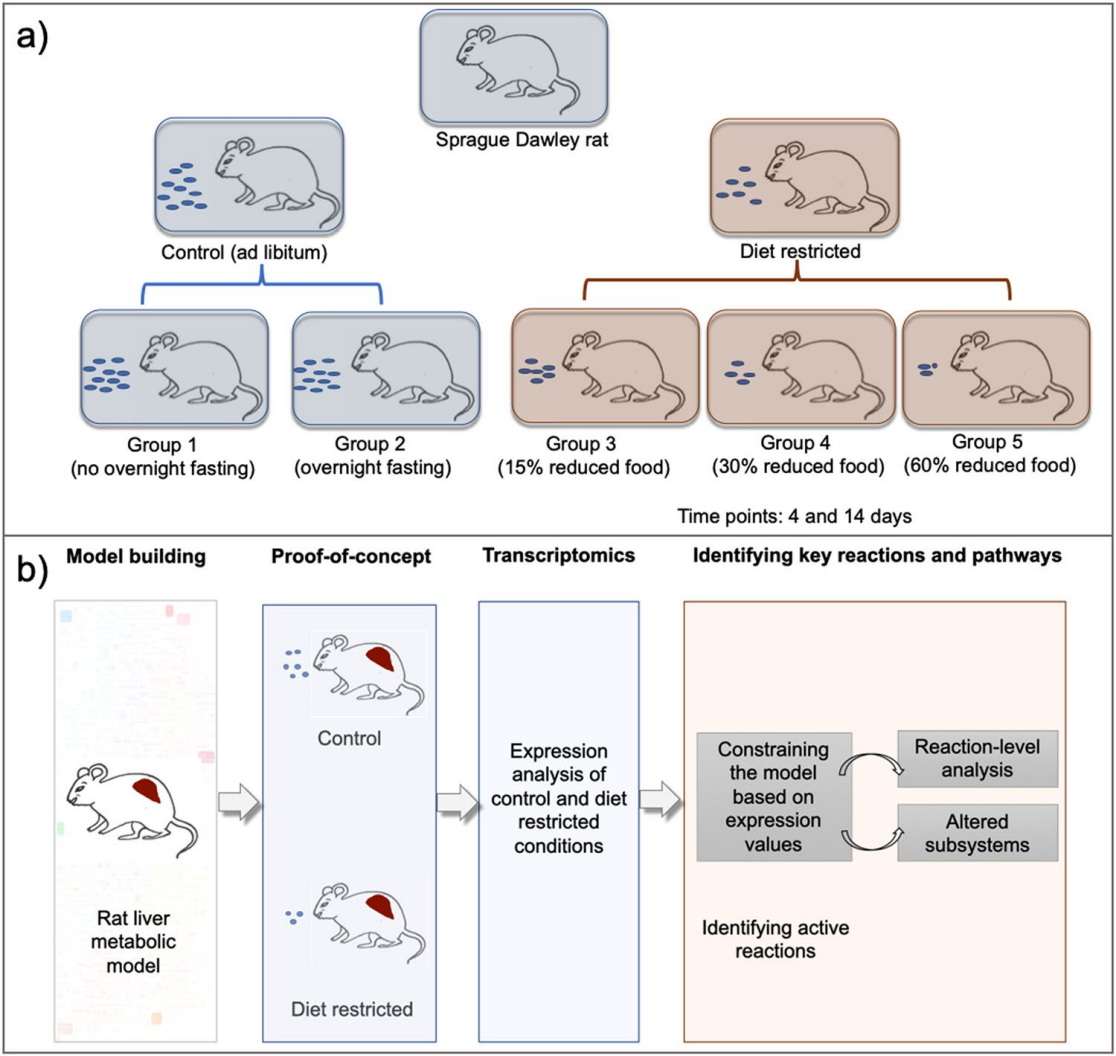
Overview of study design. (**a**) Graphical representation of the experimental groups of rats considered for studying the effect of diet restriction. The rats were divided into five groups based on their diet and the experimental measurements were done at days 4 and 14. (**b**) Graphical representation of various analyses performed in the study from reconstructing the rat liver model to identifying active reactions in diet restricted conditions.

Our next goal was to explore the utility of iRatLiver for phenotypic predictions under a controlled perturbation. Diet restriction in rats has been extensively studied in relation to a variety of medical applications, including obesity^34^, lifespan^32,49^, and drug effects^33,50^. We therefore designed a study in which we altered the diet of groups of rats and studied the resulting change in liver function through measuring gene expression (Fig. 1). A total of 50 male Sprague Dawley rats were divided into five groups and given different diets; liver samples were taken at days 4 and 14 for five rats in each group for transcriptomics analysis (see Methods for full details). From the gene expression data, we identified differentially expressed genes (DEGs) (Data S3 (Table S2)) and corresponding biological processes that were enriched for DEGs under diet-restriction conditions (Data S3 (Table S3)).

**Figure 2:**
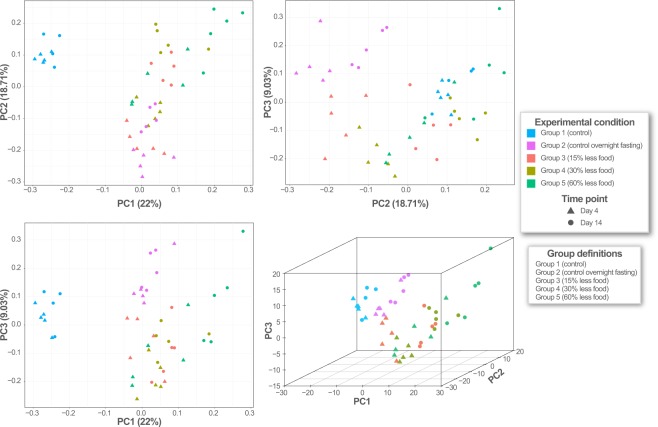
Principal component analysis (PCA) of metabolic genes present in transcriptome data. Colors correspond to experimental groups; shapes represent time points (days 4 and 14).

We then filtered the gene expression data to the set of metabolic genes to examine the effects of diet restriction on liver metabolism. We performed principal component analysis (PCA) on the normalized expression values of the metabolic genes, observing distinct clusters for the different experimental groups (Fig. 2). The control samples (with or without overnight fasting) formed a separate cluster from the diet-restricted samples, corroborating previous results that suggested the expression of metabolic genes varies under varying dietary conditions^51^. Further, the diet-restricted samples for days 4 and 14 clustered separately, indicating that the expression profile was also influenced by the duration of the diet restriction. We observed high variance in the group 5 rats (60% diet restriction), suggesting that there was a less uniform adaptive response. Ultimately, these results indicated that the underlying expression profile of the liver varied as a function of time.

Analysis of the raw transcriptomics data provided a snapshot of biological processes that might be regulated in various conditions, but our goal herein was to obtain a holistic view of how the metabolic network changed as a result of diet restriction. Thus, we integrated the transcriptomics data into the iRatLiver model using previously published methods (GIMME^12^ and E-flux^52^); the two algorithms take different approaches to integrating the expression data with the GEM, resulting in models with differing structure (see Methods). The resulting condition-specific models (i.e., a model for each group of rats in the experimental design) allowed for the exploration of how metabolic fluxes and pathway usage were altered under nutrient deprivation.

**Figure 3:**
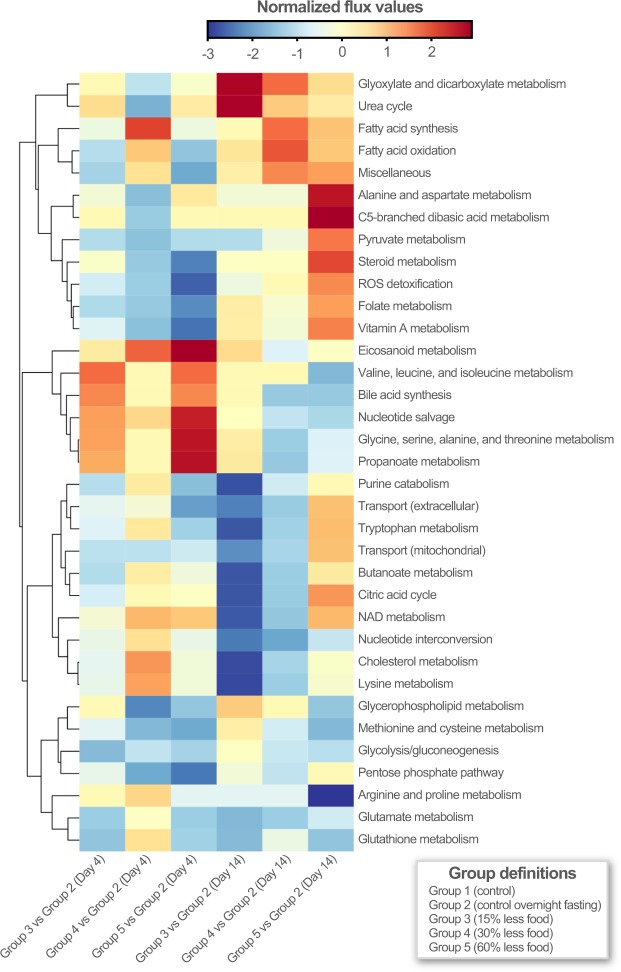
Comparison of flux states across experimental conditions. The clustergram on the left represents the extent of alteration in the subsystems for observed conditions. Rows represent various subsystems in the model and columns represent the experimental conditions. The values for each subsystem are normalized by projective decomposition.

We computed the flux state of the full metabolic network for all condition-specific models to identify reactions that carried flux in all conditions. We selected the set of reactions from GIMME and E-flux that had non-zero fluxes across a majority of conditions; this approach yielded a set of 1049 and 604 reactions that carried flux in GIMME and E-flux, respectively. The intersection of those reactions resulted in a set of 338 high-confidence reactions that were used in subsequent analysis (list of reactions provided in Data S3 (Table S5)). To obtain insight into pathway regulation under the experimental conditions, we simulated the models (optimizing for growth rate) and computed the flux state. These flux values were scaled using projective decomposition^53^, a normalization method that is part of Scale-Invariant Geometric Data Analysis (SIGDA)^54^, showing that various metabolic subsystems cluster together (Fig. 3). We observed trends in the usage of several pathways in the group 5 rats (60% less food) that were in the opposite direction when comparing the day 4 and 14 timepoints. This observation can be explained by the fact that, during initial diet restriction, the liver is able to produce glucose from the catabolism of glycogen; under long-term diet restriction (during which glycogen stores have been depleted) glucose is synthesized via gluconeogenesis from substrates such as lactate, pyruvate, glycerol, and amino acids generated in the liver or originating from extrahepatic tissues^55^.

### Understanding changes in pathway usage under diet-restricted conditions

While previous studies have explored the regulation of glycolysis, gluconeogenesis, TCA cycle and pentose phosphate metabolism^55,56^, the iRatLiver model provides the opportunity to explore these questions in the context of the metabolic network. To this end, we performed pathway-based enrichment analysis (Data S3 (Table S6)) on the genes present in altered iRatLiver model subsystems. We found that glycolysis/gluconeogenesis, the pentose phosphate pathway (PPP), fatty acid degradation, purine metabolism, and propanoate metabolism were significantly affected by diet restriction. It has been previously reported that the activity of metabolic enzymes in PPP was reduced under starvation conditions but restored through re-feeding with a high-carbohydrate diet^33^.

**Figure 4:**
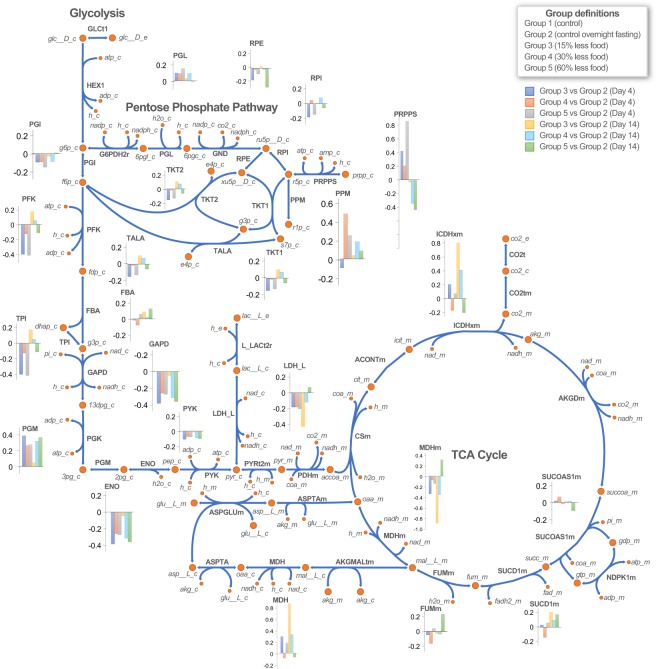
Pathway-based analysis for glycolysis, TCA cycle and pentose phosphate pathway. The bar graphs represent the distribution of fluxes in each condition shown with respect to control overnight fasting samples. Different colors correspond to different experimental conditions.

Upon deeper analysis of these subsystems, we observed that the distribution of reaction fluxes varied between conditions (Fig. 4). Several reactions relevant to starvation conditions^55^, such as L-lactate dehydrogenase (LDH_L) malate dehydrogenase (MDH), succinate dehydrogenase (SUCD1m), and phosphoribosylpyrophosphate synthetase (PRPPS), showed variations in fluxes across conditions^55^. Under starvation conditions, iRatLiver correctly predicts the synthesis of glucose through gluconeogenesis, the conversion of lactate to pyruvate (by LDH_L) to oxaloacetate in the mitochondria. The accumulation of TCA cycle intermediates in hepatocytes due to gene deletion is responsible for hepatic steatosis in dietary restricted state^55,56^. In addition to these behaviors, the iRatLiver model also predicted alterations in phosphoribosylpyrophosphate synthetase (PRPPS) under fasting conditions.

We also examined the regulation of hormones, one of the main functions of the liver, and the subsequent effect on various physiological functions in the system during diet restriction. Several genes in steroid metabolism were altered based on our metabolic analysis, namely hydroxysteroid 11-beta dehydrogenase 1 (*Hsd11b1*), hydroxysteroid (17-beta) dehydrogenase (*Hsd17b1*, *Hsd17b2*, *Hsd17b7*, *Hsd17b8*), and aldo-keto reductase family 1 (*Akr1c19*). These genes are involved in various biological processes such as glucocorticoid biosynthesis, cholesterol biosynthetic process, fatty acid biosynthesis process, oxidation-reduction process as well as response to nutrient levels. This observation suggests that hormone regulation is another important physiological change that occurs during diet restriction.

### Identifying key genes from network analysis

Finally, we attempted to understand transcriptional and metabolic variations due to diet restriction using a network-based approach. We hypothesized that the metabolic genes identified through integration of the gene expression data with the iRatLiver model were highly connected nodes that orchestrated global metabolic functionality under changing environmental conditions.

**Figure 5:**
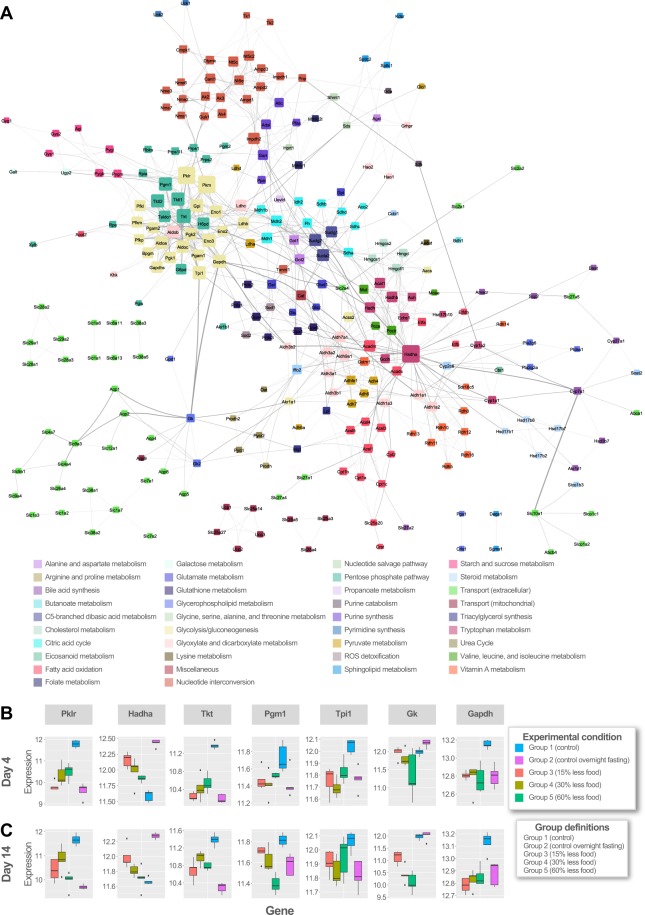
Protein expression network. (**A**) Protein interaction network representing shortlisted metabolic genes from analysis. The genes (nodes) are colored based on the subsystem they belong to and node size denotes the degree of connectivity of that node in the network. Highly connected nodes are bigger in size. The thickness of edge is decided based upon its edge betweenness in the network. (**B**) Box plots of hub nodes gene expression at Day 4. (**C**) Box plots of hub nodes gene expression at Day 14. Hub nodes were identified from network analysis. Box colors indicate experimental conditions as shown in the legend.

To test this hypothesis, we constructed a protein expression network in which genes are nodes and interactions are edges (Fig. 5A; see Methods); the interaction between nodes may be a physical binding or function association determined by putative or experimental evidence^57^. We identified the most highly connected nodes (“hub” nodes) to be pyruvate kinase (*Pkm*, *Pklr*), hydroxyacyl-CoA dehydrogenase (*Hadha*), transketolase and transketolase-like protein (*Tkt*, *Tktl1*, *Tktl2*), phosphoglucomutase (*Pgm1*), triosephosphate isomerase (*Tpi1*), and enolase (*Eno3*). We calculated the node degree distribution (the number of connections the node has to other nodes in the network) and edge betweenness (the number of shortest paths that go through an edge in the network) for the network. The edge betweenness for glycerol kinase (*Gk*) and glyceraldehyde-3-phosphate dehydrogenase (*Gapdh*) was highest, indicating that many paths in the network traverse by this edge.

Interaction networks provide interesting insights into how genes related to diverse functions work in concert to achieve broader systems-level functions^58^. We next explored how the hub nodes in our network are connected. We observed similar trends in the expression of *Hadha*, *Tpi*, *Gk*, and *Gapdh* for in the day 4 and 14 diet-restricted conditions. The trend varies for *Tkt* and *Pgm1*, indicating that these genes are differently regulated for under different diet conditions. The expression levels of *Tkt* in our study are lower when compared to control samples. *Pgm1* is expressed at higher levels in the nutrient restriction conditions than control overnight fasting, except for the 60% restricted food group at 14 days (Fig. 5B,C).

## Discussion

The liver plays an important role in regulating metabolic homeostasis by catabolizing, storing, and altering nutrients, as well as detoxifying toxic substances present in the body. Targeted perturbation experiments in model organisms allows for a systems-level characterization of the complex systems underlying liver metabolism. In particular, rats are frequently used in pharmacological and metabolic studies due to physiological similarities with humans. Here, we described a tissue-specific metabolic network model of the rat liver (iRatLiver) and used it to study diet restriction in rats. We used the iRatLiver model to compute changes in the network-level metabolic flux state of the system due to alterations in the nutritional status of 50 Sprague Dawley rats. We have provided evidence that this *in silico* model is able to provide deeper insight into the metabolic alterations in rat due to diet restriction. The results presented here have three primary implications.

First, upon diet restriction, the liver maintains homeostasis through regulation of metabolic activity across the metabolic network. We observed that several key pathways – central carbon metabolism, fatty acid degradation, purine metabolism, and propanoate metabolism – are responsible for the regulation of systems-level function under the diet restriction. Hepatocytes can use glucose and/or fatty acids as metabolic fuels, and selection of these depends on hormonal regulation and nutrient levels. In fasting or nutrient starvation conditions, hepatocytes predominantly depend on oxidation of fatty acids for energy supply^55^, an observation supported by our results herein.

Second, the liver’s response varied with respect to short- and long-term diet restriction. The survival of rats subjected to severely restricted diet indicates that the organism is able to alter its metabolism to maintain required energy levels. Through integration of gene expression data with the iRatLiver model, we were able to identify the genes and pathways responsible for the metabolic shifts that allowed the organisms to adapt to changing environmental conditions. We investigated reactions involved in carbohydrate metabolism (glycolysis/gluconeogenesis, TCA cycle, and PPP) and observed changes in the flux through phosphoribosylpyrophosphate synthetase (PRPPS) in fasting conditions. PRPPS (*Prps1*, *Prps1l1*, *Prps2*) was previously reported to be affected by amino acid depletion^55,59^, while mutations in *Prps1* have been associated with hyperuricemia, hyperuricosuria, hypotonia, and ataxia and gain-of-function mutation results in PRS-1 superactivity^60^. Our investigation of differential pathway usage under diet restriction therefore warrants further study to better characterize genetic factors that influence the varied metabolic shifts observed here in short- and long-term diet restriction.

Third, network models have the potential to provide important insights into complex liver functions. The construction of an interaction network allowed for the identification of key genes such as *Pklr*, *Hadha*, *Tkt*, *Pgm1*, *Tpi*, *Gk*, and *Gapdh* involved in metabolic regulation during diet restriction. We observed that the expression levels of *Tkt* were lower in diet restriction than in control samples. Transketolase (*Tkt*) is a key enzyme in pentose phosphate pathway and governs carbon flow. *Akt* is known to regulate *Tkt* activity, and it has been reported that caloric restriction causes downregulation of the PI3K/Akt/mTOR pathway, ultimately affecting amino acid, carbohydrate and purine metabolism^61^. Thus, we conclude that these pathways are affected under the conditions studied here. Similarly, *Pgm1* (Phosphoglucomutase) –involved in the interconversion of glucose-1-phosphate and glucose-6-phosphate – has been connected to the regulation of glycogen content during nutritional stress in the system^62^. In humans, *Pgm1* plays a role in balancing cellular demand during nutrient depletion, thus helping cell proliferation. Both *Pgm1* and *Tkt* (identified here as hub nodes) have been implicated in cancer cell proliferation and have been studied as possible therapeutic targets^61,62^. Thus, it is possible that a network approach such as the one presented here could be used in pharmaceutical studies for the identification of potential drug targets.

In this study, we used a systems approach to interrogate energy metabolism in the rat liver in a metabolic network model context. Through the integration of transcriptomics data, we generated condition-specific models that were used to compute pathway usage under different diet-restricted conditions. Our results suggest that the construction of a detailed transcriptional regulatory network of the rat liver would lead to further important insights into the effects of diet restriction, such as genes that are switched on or off during those stress conditions. We anticipate that the iRatLiver model presented here, which we are making freely available to the scientific community, will also prove useful to others in studying important physiological and biomedical questions related to obesity, aging, and pharmacology.

## Methods

### Reconstruction of an *in silico* metabolic model of rat liver

We used a previously published tissue-specific GEM of the human liver (liverCADRE^16^) for the construction of iRatLiver model. The liverCADRE model displayed improved metabolic functionality and was useful in predicting biological outcomes. The liverCADRE model consisted of 1763 reactions, 1402 metabolites, 994 unique genes and 80 subsystems^16^. Following the established protocol for reconstructing metabolic networks^35^, we identified homologous genes between human and rat using Ensembl (GRCh37)^63^ and Homologene^64^ and replaced these human genes with the corresponding *Rattus norvegicus* genes.

We incorporated information regarding known metabolic differences between rat and human (unique proteins in rat liver metabolism added to the iRatLiver model are summarized in Table 1)^38–40^. We performed protein BLAST^65^ using an e-value cut-off of 1e-30 to identify matching proteins. Next, we compared metabolic pathways in human and rat using EC2KEGG tool^66^. The list of unique proteins in rat along with the biological functions are given in Table 1. We used KEGG^67^ and BioCyc^68^ to obtain information of metabolic reactions catalyzed by these enzymes which were added to the iRatLiver model. We identified dead-end metabolites and blocked reactions in the draft reconstruction. We used reaction file containing information from Recon 2^15^, KEGG^67^ and BioCyc^68^ to fill gaps in the model.

The resulting draft reconstruction consisted of 1843 reactions, 1477 metabolites, and 988 unique genes. We then added reactions belonging to cholesterol metabolism, tryptophan metabolism, glycolysis, tyrosine metabolism and others using information from the human Recon 2 model^15^. We used the objective function from mouse metabolic model iMM1415^69^ with no modifications. The final iRatLiver model consisted of 1882 reactions, 1448 metabolites, 994 unique metabolic genes, 7 compartments, and 82 subsystems. We compared reactions and subsystems present in the human liver model (liverCADRE)^16^ and iRatLiver model and found differences in vitamin B2, vitamin C, squalene and cholesterol synthesis, fatty acid oxidation, tryptophan as well as pentose phosphate pathway.

### iRatLiver model validation

We performed two primary sets of validation for iRatLiver. First, we compared the predicted doubling time of 16.3 hours with literature values, finding that it was consistent with the reported doubling time of 16.9 hours in rat hepatocyte cell culture^37^. Second, we performed a previously used array of tests to ensure the model could compute basic functionality of the liver^16^. Specifically, we tested the model’s ability to perform gluconeogenesis, triglyceride synthesis, amino acid degradation, and ammonia and ethanol detoxification (see Data S3 (Table S4) for simulation results). We deposited iRatLiver in BioModels^70^ under the identifier MODEL1811090001. The iRatLiver model is available as SBML format (Data S2).

### Experimental design for diet restriction study in rat

We studied the effects of varying levels of nutrient deprivation on male Sprague Dawley rats, approximately 8–10 weeks of age, that were subjected to varying levels of dietary restriction (Fig. 2 and Data 3 (Table S1)). Rats were housed in groups or stable pair of compatible individuals at an AAALAC, International accredited facility and were cared for in accordance with the *Guide for the Care and Use of Laboratory Animals*, 7^th^ Edition^71^. All research protocols were reviewed and approved by the Amgen Institutional Animal Care and Use Committee. Lighting in animal holding rooms was maintained on 12:12 hr light:dark cycle, and the ambient temperature and humidity range was at 68 to 79 F and 30 to 70%, respectively. Powdered feed (Harlan Tekland rodent diet 8640) was provided either *ad libitum* (groups 1 and 2), reduced by 15% (group 3), reduced by 30% (group 4), or reduced by 60% (group 5). Reduction in food was calculated based on the average food consumption recorded for group 1 and 2 based on the previous 3 days. There were 10 rats per group; five rats per group were subjected to 4 days of dietary restriction (necropsy on day 5) and the remaining 5 per group were subjected to 14 days (euthanized on day 15). Groups 2–5 were fasted 8–12 hours overnight prior to necropsy. Standard clinical and anatomic pathology endpoints were collected and examined (not included in this study).

### Transcriptome analysis

RNA extraction from rat hepatocytes was carried out using Qiagen (Valencia, CA) RNeasy Mini kit and qiazol according to the manufacturer’s instructions to homogenize the tissues and chloroform for phase separation. Amplification was performed with Oligo dT primed RT using SSII, 2nd strand cDNA synthesis, cleanup via Qiagen MinElute. Synthesis of biotinylated cRNA using the Enzo (Farmingdale, NY) amp kit, cleanup of cRNA via Qiagen RNeasy Mini kit. Biotin-labeled aRNA products were hybridized to Affymetrix (Santa Clara, CA) GeneChip® Rat Genome 230 2.0 Arrays per manufacturer instructions provided on the product insert. Scans were carried out as per manufacturer instructions provided on the product insert. The scan files were processed using Expression Console (Affymetrix) for quality control and subsequent results were used in microarray data analysis.

All statistical analyses were executed using the R statistical computing platform (version 3.0.1). Expression data were normalized using the Robust Multi-Array Average method implemented in the Bioconductor package *affy* (version 1.38.1). The expression data was submitted in Gene Expression Omnibus (GEO)^72^ and the accession number for the data is GSE98621. A single Empirical Bayes model included in the Bioconductor package EBarrays (version 2.2.0) package, Lognormal-Normal-Modified-Variance (LNN-MV), was used to calculate differentially expressed genes. These analyses were conducted separately for each treatment group. The function *crit*.*fun*, setting FDR = 0.1, was used to calculate the minimum posterior probability required to deem a sequence representing a gene significant in each treatment group.

Normalization and fold change analysis was performed as mentioned above. The fold change values were calculated by considering the group of ad libitum, overnight fasting samples (Group 2) as control. The number of DEGs in each condition are represented in Data S3 (Table S2). The number of DEGs are higher for Day 14 conditions as compared to the others, indicating that larger number of genes are altered during high stress conditions. We identified sets of genes that are commonly up or down-regulated in various experimental groups. Enrichment analysis of these genes gave information of the pathways were significantly enriched in these conditions. We used the enrichment analysis performed by STRING v10.5^57^ (see Data S3 (Table S3)).

### Integration of transcriptomics data with iRatLiver

To identify the metabolic changes taking place in the liver, we integrated transcriptomic data with the iRatLiver model. Among available methods for integrating omics data with metabolic model^73^, we implemented two different algorithms: Gene Inactivity Moderated by Metabolism and Expression (GIMME)^12^ and E-Flux^52^. The two algorithms make differing assumptions for the integration of transcriptomics data, resulting in differing outputs and interpretations of subsequently computed physiological states.

The combination of these methods is helpful in identifying set of active reactions in condition of interest and capturing extent of flux changes and reshaping the flux cone considering the measurements of gene expression. Upon implementation of the algorithms, we computed the flux state (optimizing for growth rate) of each model separately; the reactions with zero flux were identified (reactions with a flux less than 1E-06 were denoted as carrying no flux). Reactions having non-zero fluxes were selected from the total set of reactions in the model. Only those reactions having measurable fluxes from both methods were considered active and subsequently used for analyzing reactions and determining which subsystems were perturbed under diet-restricted conditions. We considered absolute values of reaction fluxes and grouped the reactions based on their corresponding subsystems. For each subsystem, we calculated average values of reaction fluxes. Considering group 2 (control, overnight fasting) as control, we subtracted the average values calculated for each subsystem for diet-restricted conditions. Then we carried out an unbiased approach to identify subsystem differences between diet-restricted conditions. We used projective decomposition^54^ to normalize the average flux values and represented the values in clustergram in Fig. 3. The normalized flux values ranged from −3 to 2 for different subsystems. We also carried out flux variability analysis (FVA)^74^ and determined the robustness of metabolic model. The information related to altered subsystems can be extrapolated to further identify possible rewiring in the system leading to adaptation during diet-restricted state. The COBRA toolbox^75,76^ was implemented in MATLAB 2014a and academic licenses of Gurobi Optimizer v7.5 and IBM CPLEX v12.7.1 were used to solve LP and MILP problems in this study.

### Statistical analysis

Fisher’s exact test followed by a correction of the p-values for multiple testing using Benjamini-Hochberg procedure was used for pathway-based enrichment of genes identified from metabolic analysis^77^. A P-value cutoff of 0.05 was considered statistically significant in all analyses.

### Network analysis and visualization

The genes belonging to altered subsystems were used for network analysis. STRING v10.5^57^ was used for extracting confident interactions (STRING combined score >0.7) between these genes/proteins of the specific organism. The genes are represented as nodes and the interaction between nodes is called an edge. The interactions were visualized using Cytoscape^78^. We used the NetworkAnalyzer application in Cytoscape to calculate node degree distribution and edge betweenness. Nodes with highest connections in the network are defined as ‘hub’ nodes. The number of shortest paths that go through an edge in the network determines the edge betweenness.

## Supplementary information

Supplementary figure 1

Data S1

Data S3

Data S2
