# Supplementary material for: Genome-scale metabolic model of the rat liver predicts effects of diet restriction
Source: Sci Rep. 2019 Jul 8;9:9807. doi: 10.1038/s41598-019-46245-1 (PMC6614411; doi:10.1038/s41598-019-46245-1)
Supplement: Supplementary file 1 — Supplementary figure 1 [file 41598_2019_46245_MOESM1_ESM.docx]

**Genome-scale metabolic model of the rat liver predicts effects of diet restriction**

Priyanka Baloni^1^, Vineet Sangar^1^, James T Yurkovich^1^, Max Robinson^1^, Scott Taylor^2^, Christine M. Karbowski^2^, Hisham K. Hamadeh^2,†^, Yudong D. He^2^, Nathan D Price^1,^*

Email: nprice@systemsbiology.org

### Supplementary materials

Data S1: iRatLiver model in mat format

Data S2: The rat liver model is available as SBML format

Data S3: Supplementary Tables S1 through S6

**Supplementary figure**

**
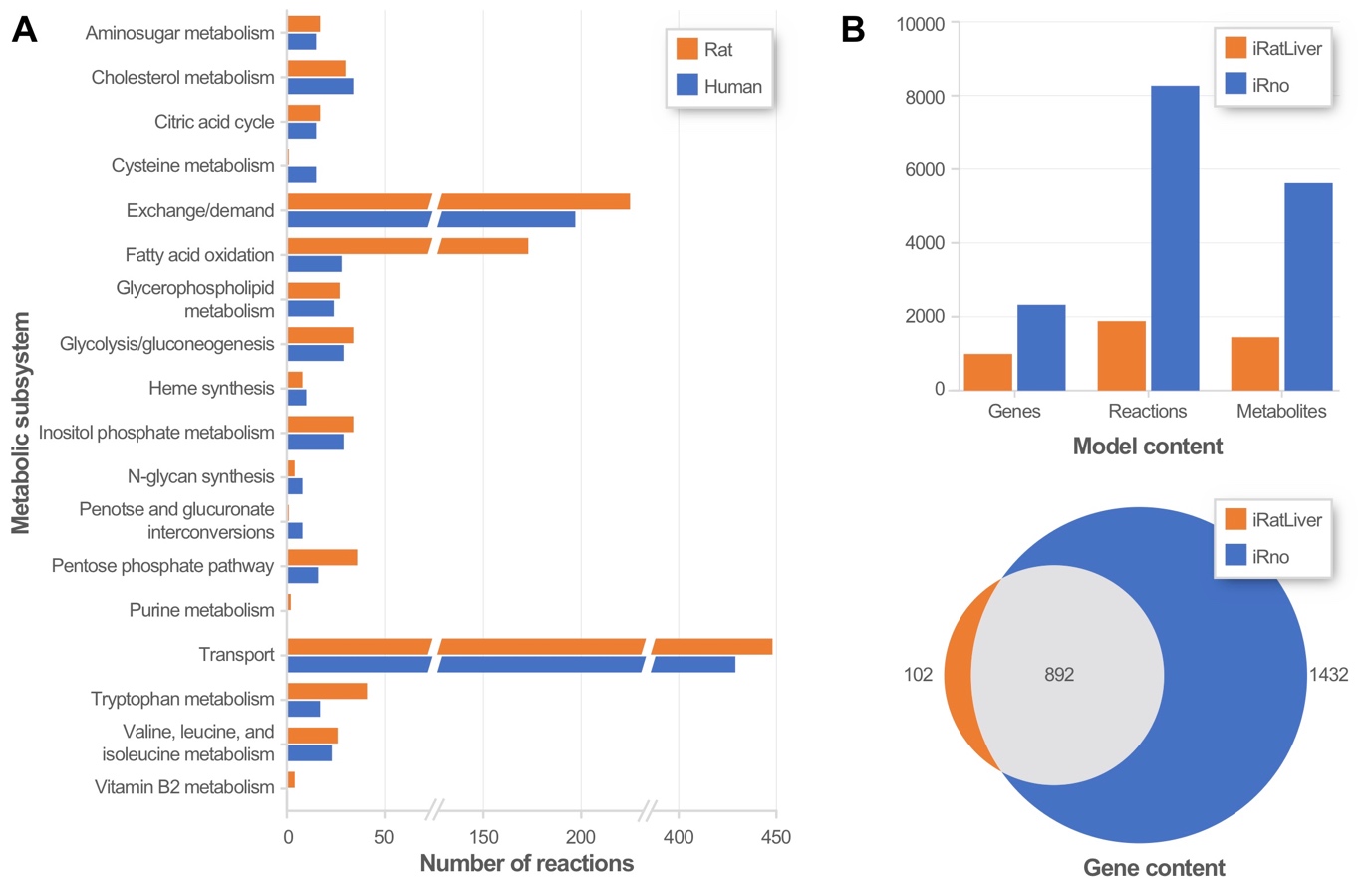
**

**Supplementary figure 1.** Overview of the iRatLiver GEM. (**A**) A comparison of the reaction content between iRatLiver and the human liver model[^16^](https://paperpile.com/c/6AsHjB/mtEV1). (**B**) A comparison of the model and gene content between iRatLiver and the global rat metabolic reconstruction iRno[^30^](https://paperpile.com/c/6AsHjB/wqsbr).
